# Why Is Tinnitus a Problem? A Qualitative Analysis of Problems Reported by Tinnitus Patients

**DOI:** 10.1177/2331216518812250

**Published:** 2018-11-28

**Authors:** Emily J. Watts, Kathryn Fackrell, Sandra Smith, Jacqueline Sheldrake, Haúla Haider, Derek J. Hoare

**Affiliations:** 1 School of Medicine, University of Nottingham, UK; 2 NIHR Nottingham Biomedical Research Centre, Hearing Sciences, Division of Clinical Neuroscience, School of Medicine, University of Nottingham, UK; 3 The Tinnitus and Hyperacusis Centre, London, UK; 4 ENT Department, Hospital Cuf Infante Santo, Nova Medical School, Lisbon, Portugal

**Keywords:** psychosocial or emotional, behavioral measures, adult or general hearing screening

## Abstract

Tinnitus is a prevalent complaint, and people with bothersome tinnitus can report any number of associated problems. Yet, to date, only a few studies, with different populations and relatively modest sample sizes, have qualitatively evaluated what those problems are. Our primary objective was to determine domains of tinnitus problem according to a large clinical data set. This was a retrospective analysis of anonymized clinical data from patients who attended a U.K. Tinnitus Treatment Center between 1989 and 2014. Content analysis was used to code and collate the responses of 678 patients to the clinical interview question “Why is tinnitus a problem?” into categories of problems (domains). We identified 18 distinct domains of tinnitus-associated problems. Reduced quality of life, tinnitus-related fear, and constant awareness were notably common problems. Clinicians need to be mindful of the numerous problem domains that might affect their tinnitus patients. Current questionnaires, as well as being measures of severity, are useful clinical tools for identifying problem domains that need further discussion and possibly measurement with additional questionnaires. The domains identified in this work should inform clinical assessment and the development of future clinical tinnitus questionnaire.

## Introduction

Often described as a ringing, whistling, or buzzing sound, tinnitus is a complex and diverse symptom, defined as the perception of a sound that has no external source (McFadden, 1982). For some, the experience of tinnitus goes beyond the *phantom* sensation of sound. It can cause problems such as insomnia, difficulty concentrating, and poor psychological well-being, ultimately decreasing symptom-specific health-related quality of life (Hall et al., 2018; Langguth & Landgrebe, 2011; Nondahl et al., 2011; Pierce et al., 2012; Stevens, Walker, Boyer, & Gallagher, 2007; Tyler & Baker, 1983). The impact of tinnitus on a person can range from mildly problematic to completely debilitating with significant social and economic consequences (Andersson, 2002; Stockdale et al., 2017). Of the 10% of the general population who experience chronic tinnitus (Landgrebe et al., 2012; McFerran & Phillips, 2007), 20% experience “clinically significant tinnitus” and seek relief from their symptoms (Henry, Jastreboff, Jastreboff, Schechter, & Fausti, 2003). However, management of tinnitus can be complex, requiring an individualized and often multifaceted approach to reduce symptoms and associated comorbidities (Decot, 2005; Department of Health, 2009; Hoare, Gander, Collins, Smith, & Hall, 2012).

Psychoacoustic estimates of tinnitus provide little information on its impact and the associated problem symptoms (Andersson, 2002; Jakes, Hallam, Chambers, & Hinchcliffe, 1985). Typically, clinicians and researchers alike rely on multiattribute self-report questionnaires to measure tinnitus severity and identify appropriate management pathways. For example, tinnitus questionnaire items can ask about particular difficulties with concentration, sleep, coping, and emotional well-being.

Negative consequences or limitations of tinnitus can be categorized into *domains* that are theoretically similar or contribute to a specific aspect of tinnitus distress or annoyance, and many tinnitus questionnaires provide measures, to varying degrees, of different problem domains associated with tinnitus. Patient interviews, to assess the effects of treatment, for example, can be structured around what are considered important domains (Andersson & Edvinsson, 2008). To belong to the same domain, consequences or limitations would have to produce a sufficiently similar effect on the patient such that questionnaire items could logically be combined to measure a specific problem caused by tinnitus. There is, however, no universal agreement on what these domains are, how many domains of tinnitus problem there are, or how these domains should be assessed (Baguley & Andersson, 2003; Hall et al., 2018). For example, the Tinnitus Handicap Questionnaire (THQ; Kuk, Tyler, Russell, & Jordan,1990) assesses handicap in relation to psychological and auditory problems, while the Tinnitus Handicap Inventory (THI; Newman, Jacobson, & Spitzer, 1996) probes problems with function, emotion, and catastrophizing. One of the more recent multiattribute questionnaires to be developed is the Tinnitus Functional Index (TFI, Meikle et al., 2012). The TFI was specifically developed to cover multiple distinct problem domains (intrusiveness, sense of control, cognition, sleep, auditory, relaxation, quality of life, and emotional impact of tinnitus), to measure tinnitus severity, and to be a responsive outcome measure (Meikle et al., 2012). More recent still, the Tinnitus Primary Function Questionnaire (Tyler et al., 2014) was developed to measure “the primary ways tinnitus impacts on a person’s life” (p. 261) with domains covering problems with sleep, hearing, concentration, and emotions.

While many of questionnaires drew heavily on previous questionnaires when selecting items or potential domains to include, the true starting point to developing any questionnaire is to identify and understand what the problems that need to be measured are. This is something that can only be answered by people who experience tinnitus and might include not only problems that arise because of tinnitus but also problems that patients confuse with or ascribe to their tinnitus (e.g., consider hearing difficulties or cognitive problems as tinnitus problems when they are more likely due to an unacknowledged hearing loss). Tyler and Baker (1983) surveyed 72 members of a Nottingham-based tinnitus self-help association asking why tinnitus was a problem to them. Respondents had an average age of 61 years (standard deviation [*SD*] = 13.1), 66% were women, 34% were men, and the mean age at onset of tinnitus was 51.9 years (range = 9–73). On average, respondents reported 4.6 difficulties due to tinnitus (range = 1–13) with fewer difficulties being reported by those who had experienced tinnitus for a longer time. The 31 problems reported were grouped into four main domains: (a) “Effects on hearing” including problems understanding speech and television, listening to the radio, appreciation of music, use of the telephone, localization of sounds, and listening to environmental sounds; (b) “Effects on lifestyle” including problems getting to sleep, persistence of tinnitus, worsening on waking, requiring or avoiding noisy situations, and conversely requiring or avoiding quiet situations, withdrawing from or avoiding friends, family problems, interference with work, difficulty learning to drive, and explaining tinnitus to others; (c) “Effects on general health” including dependence on drugs, pain and headaches, giddiness or imbalance, general ill health, ineffectiveness of drugs, tiredness, and ineffectiveness of tinnitus masker; (d) “Emotional problems” including despair, frustration and depression, annoyance, irritation and inability to relax, difficulty concentrating, confusion, insecurity, fear and worry, and consideration of suicide. The most commonly reported problems in that study were “getting to sleep,” and the “persistence of tinnitus.” Tyler and Baker acknowledge that there may have been some bias in their data set toward patients who suffer more severely as all respondents were members of a tinnitus self-help group. The same survey was subsequently used by Sanchez and Stephens (1997, 2000) to assess why tinnitus was a problem among a population of 436 tinnitus clinic patients (mean age of 57 years, range = 14–92 years, 51% women, 49% men), at baseline and at follow-up (1.5–5 years later). In this study, all respondents were patients attending a tinnitus clinic for the first time. Duration of tinnitus ranged from 1 month to 70 years, and 394 (90.1%) had some degree of hearing loss. They reported, on average, 3.78 distinct problems (range = 1–12). Younger patients (those under 50 years) reported more problems. Thirty distinct problems were reported, many of which were common to Tyler and Baker (1983), but Sanchez and Stephens (1997, 2000) determined that there were five problem domains; sleep, auditory, health, situational, and psychological problems. The most common problems were “hearing difficulties” and “sleep difficulties.” More recently, Manchaiah et al. (2017) took a deductive approach to quantify tinnitus-related problems in a population of 240 tinnitus research volunteers (mean age of 57 years, 57% men, 43% women) using the *International Classification of Functioning, Disability and Health* framework (World Health Organization, 2001). Most but not all reported problems could be classified according to the framework. The most commonly reported problems were coded as “emotional functions” and “sleep functions.”

Therefore, to date, there have been three studies evaluating problem domains associated with tinnitus, in different populations, and with relatively modest sample sizes. Here, we performed a retrospective analysis of anonymized clinical data from 678 patients who attended a Tinnitus Treatment Center in the United Kingdom. The primary aim was to identify the domains of tinnitus problem according to this large patient population.

## Materials and Methods

This study was a retrospective analysis of anonymized data that had been routinely collected from patients attending the Tinnitus and Hyperacusis Centre (London, UK) between 1989 and 2014. Data use and analysis complies with the governance procedures of the data controller (J. S.).

### Data Collection

The Tinnitus and Hyperacusis Initial Interview Form (Jastreboff & Jastreboff, 1999) was completed by an audiologist (J. S.) during the first consultation to assess each patient’s suitability for Tinnitus Retraining Therapy. The interview includes questions on tinnitus laterality, constancy, percentage awareness and annoyance, and the degree of severity, annoyance and effect on life experienced over the last month (using a 0–10 rating scales) and a single question asking patients to say “Why is Tinnitus a Problem?” in one sentence. For this question, the audiologist recorded the exact wording of patients’ responses. For example, one patient responded with “sleep disturbance is a problem, apprehension and waking sleeping.” The same questions were asked about sound tolerance and hearing loss, if indicated. A further question used a 0 to 10 rating scales to determine the degree to which each complaint (tinnitus, sound level tolerance, and hearing loss) is a life problem. For this study, we were primarily interested in the patients’ recorded responses (free-text) to the single question: *Why is Tinnitus a Problem?*

### Participants

The responses from 678 patients to the question, *Why is Tinnitus a problem?,* were analyzed.

### Content Analysis of Free-Text Data From Responses to “Why Is Tinnitus a Problem?”

Free-text responses were analyzed using a conventional content analysis approach, that is, information was collated directly from patients’ responses without imposing preexisting categories or theories (Hsieh & Shannon, 2005). Hence, the goal of content analysis here was to provide knowledge and understanding of the phenomenon under study, that is, why tinnitus is a problem, through the subjective interpretation of text data using a systematic process of coding and identifying themes in the data. Patient responses given to the question, *Why is tinnitus a problem?,* were in general short, such as “can’t control it” or “cannot work.” To avoid any misinterpretation of meaning that could occur due to a lack of context, we coded responses using only what was written rather than what was implied (Elo & Kyngäs, 2008).

Content analysis was conducted by E. J. W. (all data), K. F., S. S., and D. J. H. (each analyzed one third of responses, allocated using a random number generator: https://www.random.org/). First, authors independently analyzed their assigned data set. This involved data familiarization (reading and rereading) and the extraction of any meaningful initial units (problem codes) from each response. Meaningful units constituted parts of a sentence, a whole sentence, or a passage of text that pertains to the same topic and had to contain enough information to allow meaningful interpretation with respect to the research question. Two authors assessing the same data set met to examine and discuss their independently extracted problem codes. Each author presented their interpretations of the data, rotating who presented first in each meeting to ensure that no one author led the identification of problem codes. Any disagreements regarding these codes were discussed until consensus was reached or the other authors were consulted to reach a majority decision. To ensure consistency of coding across all pairs of authors, one author (E. J. W.) was involved in coding the entire data set. Finally, the extracted 994 problem codes were reviewed and categorized by four authors (E. J. W., K. F., S. S., and D. J. H.) into domains that were considered representative of the themes emerging from the problem codes. This was an iterative process involving (a) data familiarization of all problem codes involved all authors reading all codes and (b) identification of potential conceptual labels (domains) based on data familiarization. Any of the four authors could suggest a domain that they believed was representative of the data, but the domain was only included if all four authors agreed that it reflected the content of the problem codes; (c) allocation of each problem code to a relevant domain, continuing until all codes were allocated to a domain (Elo & Kyngäs, 2008; Graneheim & Lundman, 2004). These initial domains were then refined by all authors, checking for commonality or overlap between the content of the codes that were allocated to different domains, whether codes should be reallocated to a different domain, whether domains should be merged together, or whether there was more than one domain emerging from the group of problem codes allocated to the same domain. For example, the problem codes grouped under initial domains described as “Distraction” and “Concentration,” were sufficiently similar to combine and form a single domain, subsequently described as “Inability to Concentrate.” Initial domains described as “Anger,” “Frustration,” and “Stress” were combined to form an “Emotional Reaction to Tinnitus” domain. This iterative process continued until every problem code and domain was deemed valid, with each code only being allocated to one domain (Elo & Kyngäs, 2008).

In a final validation step, audiologist members of the British Society of Audiology tinnitus and hyperacusis special interest group (who were not authors or otherwise involved in the project) checked that the 994 extracted problem codes and domain grouping captured the essence of the raw data and represented the core themes from the data. The original raw data responses, the extracted problems codes and associated domains, and codes (25) removed for being ambiguous were examined and discussed. Each domain was discussed in turn with further refinements made through an iterative process, and the domains were finalized. One major revision to the domains involved codes initially associated with the domains “inability to relax,” “effect on social life,” and “effect on work” being combined under a new domain named “reduced quality of life.” Example codes from across those initial themes included “makes me feel tense,” “interferes with relaxation” (inability to relax); “forms a barrier between me and conversation,” “destroys relationships” (effect on social life); and “interferes with work,” “affecting my ability to do job” (effect on work). As such, the new domain encompassed tinnitus-specific health-related problems that reflect the World Health Organization (WHOQOL Group, 1995) definition of quality of life as a broad ranging concept affected in a complex way by the person’s psychological state, personal beliefs, social relationships, physical health, and their relationship to salient features of their environment.

Clinicians in the focus group felt that the codes associated with these initial domains were all simply different ways in which patients express tinnitus as having a general consequence for their quality of life. They considered the initial domain were not clinically meaningful and were all meaningfully captured as “reduced quality of life.”

### Secondary Analyses

Because many patients reported more than one tinnitus-related problem, we examined the degree to which problem codes related to different domains co-occurred within individual responses. Hierarchical cluster analysis was conducted in PAST version 3.06 (Hammer, Harper, & Ryan, 2001). In this analysis, the likelihood of reporting different set of tinnitus-related problems were estimated as Euclidean distances between problems when plotted per patient in an 18-dimensional space (representing the 18 domains in our data). We were interested in whether any of the domains identified more consistently grouped together, that is, patients with problem *x* also generally report problem *y*. This would indicate tinnitus problems likely to co-occur, or potentially that there is redundancy of a domain, that is, domain *x* and *y* are the same thing.

## Results

### Subjects

Of the 678 patients reporting reasons *why* tinnitus was a problem, the mean age was 57.2 years (*SD* = 14.0), 64% (432) were men and 36% (245) were women. Two hundred eighteen patients reported unilateral tinnitus, 302 reported bilateral tinnitus, and a further 90 reported hearing their tinnitus in their head (*n* = 610, missing = 68). Over 70% of patients (*n* = 503, missing = 83) reported fluctuations in their tinnitus, and 56% (*n* = 382, missing = 40) report sudden onset of tinnitus.

Percentage of time aware of tinnitus over the last month ranged from 0% to 100% (mean = 56%, *SD* = 30, *n* = 652). Percentage of time annoyed by tinnitus over the last month also varied from 0% to 100% of the time (mean = 39%, *SD* = 27, *n* = 580). Two hundred sixty-five patients reported previously trying treatments for tinnitus, with the average number tried being two. However, many patients had tried none (*n* = 413), and some had tried as many as five.

Of the 678 patients reporting problems, 252 patients reported hearing problems (missing = 20), yet only 54 used hearing aids. When asked to rate severity, annoyance, and effect of tinnitus on life over the last month on 0- to 10-point scales, patients averaged 4.4 (*SD* = 2.3), 6.4 (*SD* = 2.5), and 5.2 (*SD* = 2.8), respectively (*n* = 669). When asked to rate how problematic tinnitus, sound tolerance, and hearing loss were on a 0- to 10-point scale, patients averaged 5.6 (*SD* = 2.8, *n* = 649), 4.5 (*SD* = 3.0, *n* = 353), and 3.0 (*SD* = 2.9, *n* = 387), respectively.

### Data Analysis

Four hundred forty patients reported only one problem, 207 reported two problems, 30 patients reported three problems, and 1 patient reported four problems. The 994 problem codes were grouped, refined, and finalized into 18 domains of tinnitus-related problem (Table 1).

**Table 1.** 18 Domains of Tinnitus Handicap, the Number of, and Examples of Relevant Codes.

Domain number	Domain name	*n* codes in sample	Example codes
1	Reduced quality of life	125	Spoiling life; interferes with everything, functional through emotional
2	Fear	107	Scares the life out of me; fear of it always being there in future; I perceive it as a threat
3	Constant awareness	99	The focus of my life; always there; constant sound
4	Annoyance	87	Annoying; constant irritation; irritability; noise is really bothering me
5	Inability to concentrate	81	Wants all my attention; cannot concentrate
6	Loss of quiet	72	Feel it will never be quiet again; impacts my quiet time
7	Feeling deficient due to tinnitus	63	Wants to be perfect; feels damaged. Based on own measure of before and after.
8	Loss of control	53	No choice; can’t do anything about it; don’t have control over it; a problem I cannot solve
9	Effect on sleep and alertness	50	Difficult to sleep; wake up tired
10	Emotional consequences of tinnitus	49	Very distressing; makes me feel as if I’m falling apart
11	Effect on listening	44	Affects hearing of what’s around; noise gives me less opportunity to hear
12	Emotional reaction to tinnitus	39	Stresses me out; driving me mad
13	Loss of sense of self	31	There and it wasn’t before; changing my personality
14	Physical effects of tinnitus	22	Makes me feel unwell; tiring
15	Unpleasantness of percept	22	Too loud to handle; sharp frequency
16	Intrusiveness	19	Constantly invasive; intrusion in my head
17	Need for knowledge	17	Why do I have it?; will it always be there?
18	Loss of peace	14	Shattered my peace; stops me finding peace

**Figure 1.**
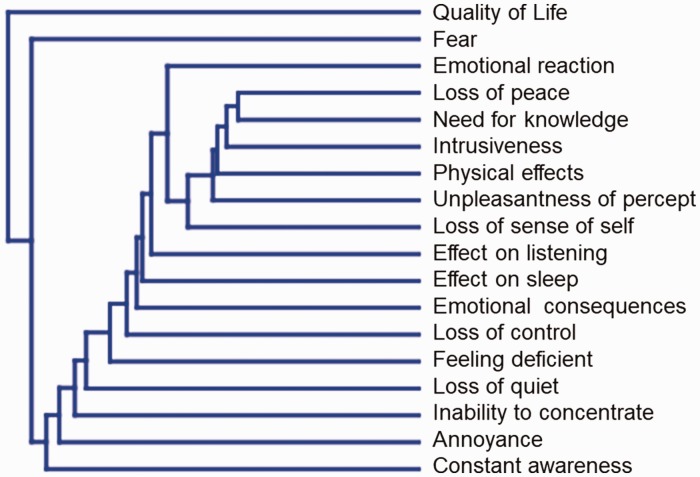
Cluster analysis indicating relatedness of tinnitus problems within the responses from individual patients.

The domains “Reduced quality of life” and “Fear” included the highest numbers of problem codes (125 and 107, respectively), while “Need for knowledge” and “Loss of peace” had the fewest (17 and 14 codes, respectively; Table 1). Cluster analysis revealed an apparent independence of the problem domains we defined (Figure 1). Euclidean distances between domains when plotted in an 18-dimensional space are given in Supplemental Information 1; the smaller the “distance” between domains the more common it was for these domains to be reported together by the same patient. Hence, “Loss of peace” and “Need for knowledge” were most commonly reported together, whereas “Quality of life” and “Constant awareness” were least often reported together.

## Discussion

In this largest study of its kind to date, we examined why tinnitus is reported as a problem in a clinical population. A retrospective analysis of data from 678 patients attending a tinnitus and hyperacusis clinic, whom identified one or more reasons why tinnitus was a problem, led us to identify 18 distinct problem domains.

### Strengths and Limitations

Due to the subjective nature of qualitative research, the involvement of four analysts, and our stepped process of identifying and verifying domains, supports there being rigor in the analytical process. The sample in this study is representative of a typical tinnitus patient population in terms of age and gender. However, patients were attending a private clinic, which would indicate at least some patients are from a more affluent socioeconomic group than may have been represented if this study had been completed through a National Health Service clinic, for example, where care is free at point of access (some patients in our sample may have previously used or discounted National Health Service care). Variables such as tinnitus severity may differ between our sample and that in other populations and influence what is reported as a problem. If, for example, our sample had more severe tinnitus, then they would more likely have comorbid anxiety or depression and potentially ascribe additional problems to their tinnitus (Bhatt, Bhattacharyya, & Harris, 2017). Furthermore, the sample includes clinical data collected over nearly three decades. Given advances in our understanding of tinnitus and the support and informational resources now available, it may be that some problem domains are much less an issue than they were 30 years ago.

A limitation of the Tinnitus and Hyperacusis Initial Interview Form may be that it is not a self-report questionnaire. It was completed by the clinician during initial consultation, who recorded the exact wording of the patients’ responses. However, the consultations are not audio recorded and as consequence the free-text responses cannot be verified. That being said, all forms were completed by the same clinician, so there is no discrepancy in user completion. There was also a small amount of demographic data missing.

It is acknowledged that reporting a reason why tinnitus is a problem does not imply causation or any relation between tinnitus and the problem reported at all. It may simply be that the problem is ascribed or confused with tinnitus. A full clinical evaluation is therefore indicated to disambiguate, for example, hearing problems due to hearing loss and those incorrectly ascribed by the patient to their tinnitus (Henry et al., 2015). That may well be an issue in the current data set given only a minority of those who reported hearing problems also reported using hearing aids.

### Why Tinnitus Is a problem?

Of the 18 problem domains identified, the most common, collectively accounting for 53% of the total codes, were “Reduced quality of life,” “Fear,” “Constant Awareness” “Annoyance,” and “Inability to Concentrate.” Here, we discuss evidence of those domains in the literature.

“Reduced quality of life” emerged here as the commonest problem domain. This is in part due to its breadth; based on the clinician focus group, it came to include a number of initial smaller domains relating to the effects of tinnitus on work, social life, and relaxation. It refers therefore to a general degradation or “spoiling” of the quality of daily activities and experiences, relating most closely to the “Effects on lifestyle” domain described by Tyler and Baker (1983). As a construct, quality of life is widely discussed in the tinnitus literature (Erlandsson & Hallberg, 2000; Härter, Maurischat, Weske, Laszig, & Berger, 2004; Nondahl et al., 2007). As a term, it is broad ranging, from use to mean general well-being of individuals and society, to health specific, where for tinnitus, it is used to describe tinnitus questionnaires quite generally (i.e., as measures of tinnitus-specific health-related quality of life). However, it also appears as a distinct construct of tinnitus problem as a subscale in the TFI, which has been shown to measure a different construct to general quality of life (Fackrell, Hall, Barry, & Hoare, 2016). In a similar fashion to how this domain emerged in this study, in developing the TFI Quality of Life subscale, domains initially considered distinct termed “Social Distress,” “Leisure,” and “Work” domains were grouped to form a single broad subscale (Meikle et al., 2012). As a domain, therefore, quality of life provides a subscale but not one that in itself is, for example, indicative of the need for a particular intervention. Rather, it is only useful as general marker of tinnitus problem level.

“Fear” is a domain that has previously been underrepresented in analyses, questionnaires, and therapies. It includes, for example, a fear of the tinnitus itself, or a fear for a future with tinnitus, or fears of activities or sounds somehow making the tinnitus worse. Fears of the unknown are more specifically considered anxieties related to imprecise or unknown threats (Öhman, 2008). However, here we need to distinguish fear as a tinnitus domain away from the construct of anxiety. Although reported as an issue by 12 of the 72 respondents (16.6%), fear is not featured as an individual problem within Tyler and Baker (1983). It is included within their “Emotional Problems” domain, as part of the problem: “Insecurity, Fear and Worry.” Sanchez and Stephens (1997, 2000), who build on Tyler and Baker’s work, do not report fear as a specific problem domain. None of the tinnitus questionnaires mentioned previously explicitly use questions that would measure tinnitus-related fear. These questionnaires were developed prior to this study and are based on the domains established by earlier works, which similarly do not single out problems related to fear. More recently developed tinnitus questionnaires either provide a composite measure of tinnitus-related fear, the Fear of Tinnitus Questionnaire (Cima, Crombez, & Vlaeyen, 2011), and the Tinnitus Fear-Avoidance Cognitions and Behaviors Scale (Kleinstäuber et al., 2013) or include a number of relevant items, for example, the Self-efficacy for Tinnitus Management Questionnaire (Smith & Fagelson, 2011).

Fear is proposed to be a key factor in the maintenance of chronic tinnitus distress by Cima et al. (2011), as measured by a self-devised *Fear of Tinnitus Questionnaire*, developed for their study and yet to be validated. Cima et al. (2011) proposed a fear-avoidance model for tinnitus based on a model originally proposed for pain (Vlaeyen & Linton, 2000). This model predicts that the less tinnitus is experienced as a threat, the more accepted it becomes. Based on this concept, Cima et al. (2011) developed a cognitive behavioral therapy-based treatment that includes elements of Tinnitus Retraining Therapy, with the aims of decreasing patients’ fear of tinnitus and correcting their “dysfunctional beliefs” (p. 1958) about tinnitus. While their treatment has shown some benefit for patients in terms of reduced handicap even though the tinnitus percept might not have changed, the precise mechanism of benefit, for example, extinction of fear, cognitive restructuring, requires evaluation using valid and specific questionnaire measures.

“Constant awareness” of tinnitus as a problem is not explicitly featured in previous studies, perhaps because awareness is considered to be an *implicit* problem. However, the codes in our study demonstrated that being aware of their tinnitus was, for many patients, their main issue. Reflective of this, there has been a growing recent interest in mindfulness and acceptance based interventions for tinnitus management (Hesser, Westin, Hayes, & Andersson, 2009).

Awareness is captured to more or less a degree by different tinnitus questionnaires. For example, the THI asks, “Do you feel as though you cannot escape your tinnitus?,” whereas the TFI includes an explicit question about percentage awareness over the last week within its Intrusiveness subscale. In clinical studies, awareness is sometimes captured through a tinnitus diary over a specified time period (Kröner-Herwig, Frenzel, Fritsche, Schilkowsky, & Esser, 2003; Zachriat & Kröner-Herwig, 2004) or more simply on a percentage of awake time awareness rating (e.g., Molini et al., 2010). Patient reports of awareness in our study were more related to the constancy of awareness; that it was inescapable, or permanent, although there were responses that simply stated tinnitus was a problem because “I’m aware of it.” Interestingly in the study by Molini et al. (2010), there was an improvement in their primary measure for most patients after treatment yet percentage awareness did not change. There may be a disparity therefore between “awareness” and what might make tinnitus clinically bothersome for an individual. Consequently, in terms of awareness, it would seem best to include a measure of it in the context of a multiattribute questionnaire rather than relying on it alone as a measure of handicap or benefit.

“Annoyance” in this study was determined from codes ranging from tinnitus being “a little annoying” to being a “constant irritation.” It was included with “Emotional Problems,” as part of “Annoyance, irritation and inability to relax” by Tyler and Baker (1983). Interestingly, Sanchez and Stephens (1997, 2000) did not report annoyance as one of their problem domains. Annoyance does feature in a number of clinical questionnaires. One of the questions of the THQ asks (to what degree) “Tinnitus makes me feel annoyed” (Kuk et al., 1990). Item 3 in the TFI asks, “What percentage of your time awake were you annoyed by your tinnitus?” This item is pooled with items related to awareness and tinnitus loudness in the “Intrusiveness” subscale (Meikle et al., 2012). The THI (Newman et al., 1996) does not mention annoyance specifically but does question “irritability” and “upset” due to tinnitus.

In the literature, annoyance is acknowledged as an important issue. Hiller and Goebel (2006) comment that tinnitus annoyance contributes heavily to the level of tinnitus severity. Andersson (2002) suggests both psychological and educational aspects to a treatment plan to tackle annoyance. In this study, annoyance was quite consistently coded as tinnitus being annoying or initiating, suggesting it is an important construct to measure routinely and specifically in clinical practice and research.

“Inability to concentrate” was, as with other domains, reported across a spectrum from “mildly distracting to permanent distraction” and “can’t think about anything else.” Problems of concentration and confusion are grouped under “Emotional Problems” by Tyler and Baker (1983). Concentration problems ranked as the most common problem in both studies by Sanchez and Stephens (1997, 2000). A number of questionnaires provide a measure of concentration problem. Conrad et al. (2015) reports the recently developed Tinnitus Cognitions Scale (T-Cog) which they found to provide subscale measures of “tinnitus-related catastrophic thinking” and “tinnitus-related avoidance cognitions.” The THQ asks (to what degree): “I cannot concentrate because of tinnitus.” The THI asks, *Because of your tinnitus, is it difficult for you to concentrate?*, and *Because of your tinnitus, is it difficult for you to read?* which could be used as further insight into concentration issues. The TFI Cognitive subscale provides a multiitem measure of this seemingly important domain (Meikle et al., 2012). More recently, Bankstahl and Görtelmeyer (2013) published a self-report tinnitus questionnaire specifically to measure the degree of cognitive failures and mishaps that are relevant to performing adequately in daily life. This questionnaire is yet to be widely used.

A number of studies have also explored associations between tinnitus and performance on *behavioral* measures of memory (Hallam, McKenna, & Shurlock, 2004; Rossiter, Stevens, & Walker, 2006; Stevens et al., 2007) or attention (Hallam et al., 2004; McKenna & Hallam, 1999; McKenna, Hallam, & Shurlock, 1995; Stevens et al., 2007). These studies provide mixed evidence of any association and have particular methodological limitations that make further research warranted (Mohamad, Hoare, & Hall, 2016). As a result, a reliable link between performance-based and questionnaire-based measures is yet to be determined.

## Conclusions

This study points to 18 distinct domains of tinnitus problem that need to be considered in tinnitus assessment and in the development of assessment tools or questionnaire measures of the impact of tinnitus. A single questionnaire of 18 domains would require at least 54 items (Meikle et al., 2012); however, this would not be practical for use in every clinical or research situation. Furthermore, patients will not report problems in all 18 domains at preintervention assessment for example, making the same questions redundant in a postintervention assessment. One possible action is to remove any domains considered irrelevant to an individual at preintervention, so they are not measured at posttreatment assessment (Tyler et al., 2014). An effective assessment needs to allow patients to express exactly what problems they are having, then more domain-specific questionnaires such as the Fear of Tinnitus Questionnaire (Cima et al., 2011) can be selected.

Current tinnitus questionnaires provide measures of various combinations of the domains identified here, but no single questionnaire covers all domains. A comprehensive measurement of all possible domains identified herein would require a combination of tinnitus questionnaires to be used.

## Supplementary Material

Supplementary material

## References

[bibr1-2331216518812250] Andersson G. (2002) Psychological aspects of tinnitus and the application of cognitive-behavioural therapy. Clinical Psychology Review 22(22): 971–990. 10.1016/s0272-7358(01)00124-6 12238249

[bibr2-2331216518812250] Andersson G. Edvinsson E. (2008) Mixed feelings about living with tinnitus: A qualitative study. Audiological Medicine 6(1): 48–54.

[bibr3-2331216518812250] Baguley D. M. Andersson G. (2003) Factor analysis of the Tinnitus Handicap Inventory. American Journal of Audiology 12(1): 31–34. 12894865 10.1044/1059-0889(2003/007)

[bibr4-2331216518812250] Bankstahl U. S. Görtelmeyer R. (2013) Measuring subjective complaints of attention and performance failures—Development and psychometric. Health & Quality of Life Outcomes 11(83): 86. 23714398 10.1186/1477-7525-11-86 PMC3674948

[bibr5-2331216518812250] Bhatt J. M. Bhattacharyya N. Lin H. W. (2017) Relationships between tinnitus and the prevalence of anxiety and depression. The Laryngoscope 127(2): 466–469. 27301552 10.1002/lary.26107 PMC5812676

[bibr6-2331216518812250] Cima R. F. F. Crombez G. Vlaeyen J. W. S. (2011) Catastrophizing and fear of tinnitus predict quality of life in patients with chronic tinnitus. Ear and Hearing 32(5): 634–641. 21399500 10.1097/AUD.0b013e31821106dd

[bibr7-2331216518812250] Conrad I. Kleinstäuber M. Jasper K. Hiller W. Andersson G. Weise C. (2015) The changeability and predictive value of dysfunctional cognitions in cognitive behavior therapy for chronic tinnitus. International Journal of Behavioral Medicine 22(2): 239–250. 25031187 10.1007/s12529-014-9425-3

[bibr8-2331216518812250] Decot, E. (2005). Therapeutic methods for psychosomatic disorders in oto-rhino-laryngology. *GMS Current Topics in Otorhinolaryngology—Head and Neck Surgery, 4*, Doc 21. PMC3200998 22073069

[bibr9-2331216518812250] Department of Health. (2009). *Provision of services for adults with tinnitus: A good practice guide*. Retrieved from http://webarchive.nationalarchives.gov.uk/20130107105354/http:/www.dh.gov.uk/prod_consum_dh/groups/dh_digitalassets/documents/digitalasset/dh_093810.pdf.

[bibr10-2331216518812250] Elo S. Kyngäs H. (2008) The qualitative content analysis process. Journal of Advanced Nursing 62(1): 107–115. 18352969 10.1111/j.1365-2648.2007.04569.x

[bibr11-2331216518812250] Erlandsson S. I. Hallberg L. R. (2000) Prediction of quality of life in patients with tinnitus. British Journal of Audiology 34(1): 11–19. 10759074 10.3109/03005364000000114

[bibr12-2331216518812250] Fackrell K. Hall D. A. Barry J. G. Hoare D. J. (2016) Psychometric properties of the Tinnitus Functional Index (TFI): Assessment in a UK research volunteer population. Hearing Research 335: 220–235. 26415998 10.1016/j.heares.2015.09.009 PMC5708524

[bibr13-2331216518812250] Graneheim U. H. Lundman B. (2004) Qualitative content analysis in nursing research: Concepts, procedures and measures to achieve trustworthiness. Nurse Education Today 24(2): 105–112. 14769454 10.1016/j.nedt.2003.10.001

[bibr14-2331216518812250] Hall D. A. Fackrell K. Li A. B. Thavayogan R. Smith S. Kennedy V. Lourenço V. M. (2018) A narrative synthesis of research evidence for tinnitus-related complaints as reported by patients and their significant others. Health and Quality of Life Outcomes 16(1): 61. 29642913 10.1186/s12955-018-0888-9 PMC5896078

[bibr15-2331216518812250] Hallam R. S. McKenna L. Shurlock L. (2004) Tinnitus impairs cognitive efficiency. International Journal of Audiology 43(4): 218–226. 15250126 10.1080/14992020400050030

[bibr16-2331216518812250] Hammer Ø. Harper D. A. T. Ryan P. D. (2001) Paleontological statistics software: Package for education and data analysis. Palaeontologia Electronica 4.

[bibr17-2331216518812250] Härter M. Maurischat C. Weske G. Laszig R. Berger M. (2004) Psychological stress and impaired quality of life in patients with tinnitus. HNO 52(2): 125–131. 14968314 10.1007/s00106-003-0889-8

[bibr18-2331216518812250] Henry J. A. Griest S. Zaugg T. L. Thielman E. Kaelin C. Galvez G. Carlson K. F. (2015) Tinnitus and hearing survey: A screening tool to differentiate bothersome tinnitus from hearing difficulties. American Journal of Audiology 24(March): 66–77. 25551458 10.1044/2014_AJA-14-0042 PMC4689225

[bibr19-2331216518812250] Henry J. A. Jastreboff M. M. Jastreboff P. J. Schechter M. A. Fausti S. A. (2003) Guide to conducting tinnitus retraining therapy initial and follow-up interviews. Journal of Rehabilitation Research and Development 40(2): 157–177. 15077641

[bibr20-2331216518812250] Hesser H. Westin V. Hayes S. C. Andersson G. (2009) Clients’ in-session acceptance and cognitive defusion behaviors in acceptance-based treatment of tinnitus distress. Behaviour Research and Therapy 47(6): 523–528. 19268281 10.1016/j.brat.2009.02.002

[bibr21-2331216518812250] Hiller W. Goebel G. (2006) Factors influencing tinnitus loudness and annoyance. Archives of Otolaryngology—Head & Neck Surgery 132(12): 1323–1330. 17178943 10.1001/archotol.132.12.1323

[bibr22-2331216518812250] Hoare D. J. Gander P. E. Collins L. Smith S. Hall D. A. (2012) Management of tinnitus in English NHS audiology departments: An evaluation of current practice. Journal of Evaluation in Clinical Practice 18(2): 326–334. 21087449 10.1111/j.1365-2753.2010.01566.x PMC3489049

[bibr23-2331216518812250] Hsieh H.-F. Shannon S. E. (2005) Three approaches to qualitative content analysis. Qualitative Health Research 15(9): 1277–1288. 16204405 10.1177/1049732305276687

[bibr24-2331216518812250] Jakes S. C. Hallam R. S. Chambers C. Hinchcliffe R. (1985) A factor analytical study of tinnitus complaint behaviour. Audiology 24(3): 195–206. 4004646 10.3109/00206098509070103

[bibr25-2331216518812250] Jastreboff, M. M., & Jastreboff, P. J. (1999). Questionnaires for assessment of the patients and treatment outcome. In Jonathan W. P. Hazell (Ed.), *Proceedings of the Sixth International Tinnitus Seminar* (pp. 487–490). London, UK: Tinnitus and Hyperacusis Centre.

[bibr26-2331216518812250] Kleinstäuber M. Jasper K. Schweda I. Hiller W. Andersson G. Weise C. (2013) The role of fear-avoidance cognitions and behaviors in patients with chronic tinnitus. Cognitive Behaviour Therapy 42(2): 84–99. 23199238 10.1080/16506073.2012.717301

[bibr27-2331216518812250] Kröner-Herwig B. Frenzel A. Fritsche G. Schilkowsky G. Esser G. (2003) The management of chronic tinnitus: Comparison of an outpatient cognitive-behavioral group training to minimal-contact interventions. Journal of Psychosomatic Research 54(4): 381–389. 12670617 10.1016/s0022-3999(02)00400-2

[bibr28-2331216518812250] Kuk F. K. Tyler R. S. Russell D. Jordan H. (1990) The psychometric properties of a Tinnitus Handicap Questionnaire. Ear & Hearing 11(6): 434–445. 2073977 10.1097/00003446-199012000-00005

[bibr29-2331216518812250] Landgrebe M. Azevado A. Baguley D. Bauer C. Cacace A. Coelho C. Langguth B. (2012) Methodological aspects of clinical trials in tinnitus: A proposal for an international standard. Journal of Psychosomatic Research 73(2): 112–121. 22789414 10.1016/j.jpsychores.2012.05.002 PMC3897200

[bibr30-2331216518812250] Langguth B. Landgrebe M. (2011) Tinnitus and depression. Textbook of Tinnitus 2975(May): 493–498.

[bibr31-2331216518812250] Manchaiah V. Beukes E. W. Granberg S. Durisala N. Baguley D. M. Allen P. M. Andersson G. (2017) Problems and life effects experienced by tinnitus research study volunteers: An exploratory study using the ICF classification. Journal of the American Academy of Audiology. *Advance online publication*,1–12. 10.3766/jaaa.17094 30479266

[bibr32-2331216518812250] McFadden D. (1982) Tinnitus: Facts, theories, and treatment, Washington, DC: National Academics Press. 25032460

[bibr33-2331216518812250] McFerran D. J. Phillips J. S. (2007) Tinnitus. Journal of Laryngology and Otology 121(3): 201–208. 16995967 10.1017/S0022215106002714

[bibr34-2331216518812250] McKenna, L., & Hallam, R. (1999). A neuropsychological study of concentration problems in tinnitus patients. In Jonathan W. P. Hazell (Ed.), *Proceedings of the Sixth International Tinnitus Seminar* (pp. 108–113). Cambridge, UK: Tinnitus and Hyperacusis Centre.

[bibr35-2331216518812250] McKenna, L., Hallam, R. S., & Shurlock, L. (1995). Cognitive functioning in tinnitus patients. In Gloria E. Reich and Jack A. Vernon (Ed.), *Proceedings of the Fifth International Tinnitus Seminar* (Vol. 1996, pp. 589–595). Portland, AQ: American Tinnitus Association.

[bibr36-2331216518812250] Meikle M. B. Henry J. A. Griest S. E. Stewart B. J. Abrams H. B. McArdle R. Vernon J. A. (2012) The Tinnitus Functional Index: Development of a new clinical measure for chronic, intrusive tinnitus. Ear & Hearing 33(2): 153–176. 22156949 10.1097/AUD.0b013e31822f67c0

[bibr37-2331216518812250] Mohamad N. Hoare D. J. Hall D. A. (2016) The consequences of tinnitus and tinnitus severity on cognition: A review of the behavioural evidence. Hearing Research 332: 199–209. 26523370 10.1016/j.heares.2015.10.001

[bibr38-2331216518812250] Molini E. Faralli M. Calenti C. Ricci G. Longari F. Frenguelli A. (2010) Personal experience with tinnitus retraining therapy. European Archives of Oto-Rhino-Laryngology 267(1): 51–56. 19543742 10.1007/s00405-009-1015-7

[bibr39-2331216518812250] Newman C. W. Jacobson G. P. Spitzer J. B. (1996) Development of the Tinnitus Handicap Inventory. Archives of Otolaryngology—Head and Neck Surgery 122(2): 143–148. 8630207 10.1001/archotol.1996.01890140029007

[bibr40-2331216518812250] Nondahl D. M. Cruickshanks K. J. Dalton D. S. Klein B. E. Klein R. Schubert C. R. Wiley T. L. (2007) The impact of tinnitus on quality of life in older adults. Journal of the American Academy of Audiology 18(3): 257–266. 17479618 10.3766/jaaa.18.3.7

[bibr41-2331216518812250] Nondahl D. M. Cruickshanks K. J. Huang G. H. Klein B. E. K. Klein R. Javier N. F. Tweed T. S. (2011) Tinnitus and its risk factors in the Beaver Dam Offspring Study. International Journal of Audiology 50(5): 313–320. 21309642 10.3109/14992027.2010.551220 PMC3073029

[bibr42-2331216518812250] Öhman, A. (2008). Fear and anxiety. In M. Lewis, J. M. Haviland-Jones, & L. Feldman Barrett (Eds.), *Handbook of Emotions* (pp. 709–729). New York, NY: The Guilford Press.

[bibr43-2331216518812250] Pierce K. J. Kallogjeri D. Piccirillo J. F. Garcia K. S. Nicklaus J. E. Burton H. (2012) Effects of severe bothersome tinnitus on cognitive function measured with standardized tests. Journal of Clinical and Experimental Neuropsychology 34(2): 126–134. 22168528 10.1080/13803395.2011.623120 PMC3313073

[bibr44-2331216518812250] Rossiter S. Stevens C. Walker G. (2006) Tinnitus and its effect on working memory and attention. Journal of Speech, Language, and Hearing Research 49(1): 150–160. 10.1044/1092-4388(2006/012) 16533080

[bibr45-2331216518812250] Sanchez L. Stephens S. D. G. (1997) A tinnitus problem questionnaire in a clinic population. Ear & Hearing 18: 210–217. 9201456 10.1097/00003446-199706000-00004

[bibr46-2331216518812250] Sanchez L. Stephens S. D. G. (2000) Perceived problems of tinnitus clinic clients at long-term follow up. Journal of Audiological Medicine 9(2): 94–103.

[bibr47-2331216518812250] Smith S. L. Fagelson M. (2011) Development of the self-efficacy for tinnitus management questionnaire. Journal of the American Academy of Audiology 22(7): 424–440. 21993049 10.3766/jaaa.22.7.4

[bibr48-2331216518812250] Stevens C. Walker G. Boyer M. Gallagher M. (2007) Severe tinnitus and its effect on selective and divided attention. International Journal of Audiology 46(5): 208–216. 17487668 10.1080/14992020601102329

[bibr49-2331216518812250] Stockdale D. McFerran D. Brazier P. Pritchard C. Kay T. Dowrick C. Hoare D. J. (2017) An economic evaluation of the healthcare cost of tinnitus management in the UK. BMC Health Services Research 17(1): 577. 28830503 10.1186/s12913-017-2527-2 PMC5567641

[bibr50-2331216518812250] Tyler R. Ji H. Perreau A. Witt S. Noble W. Coelho C. (2014) Development and validation of the Tinnitus Primary Function Questionnaire. American Journal of Audiology 23: 260–272. 24811293 10.1044/2014_AJA-13-0014 PMC12768218

[bibr51-2331216518812250] Tyler R. S. Baker L. J. (1983) Difficulties experienced by tinnitus sufferers. Journal of Speech and Hearing Disorders 48(May): 150–154. 6621006 10.1044/jshd.4802.150

[bibr52-2331216518812250] Vlaeyen J. W. S. Linton S. J. (2000) Fear-avoidance and its consequences in chronic musculoskeletal pain: A state of the art. Pain 85(3): 317–332. 10781906 10.1016/S0304-3959(99)00242-0

[bibr53-2331216518812250] WHOQOL Group (1995) The World Health Organization quality of life assessment (WHOQOL): Position paper from the World Health Organization. Social Science & Medicine 41(10): 1403–1409. 8560308 10.1016/0277-9536(95)00112-k

[bibr54-2331216518812250] World Health Organization (2001) International classification of functioning, disability and health, Geneva, Switzerland: Author.

[bibr55-2331216518812250] Zachriat C. Kröner-Herwig B. (2004) Treating chronic tinnitus: Comparison of cognitive-behavioural and habituation-based treatments. Cognitive Behaviour Therapy 33(4): 187–198. 15625793 10.1080/16506070410029568

