# Supplementary material for: Why Is Tinnitus a Problem? A Qualitative Analysis of Problems Reported by Tinnitus Patients
Source: Trends Hear. 2018 Nov 28;22:2331216518812250. doi: 10.1177/2331216518812250 (PMC6277754; doi:10.1177/2331216518812250)
Supplement: Supplementary material [file R1.pdf]

**SUPPLEMENTAL INFORMATION 1. Euclidian distances between problems in an 18-dimensional space**

| | Constant Awareness | Emotional Reaction | Loss of Peace | Loss of Quiet | Emotional Consequences | Feeling Imperfect | Inability to Concentrate | Loss of Control | Fear | Effect on Listening | Quality of Life | Physical Effects of Tinnitus | Intrusiveness | Effect on Sleep | Need for Knowledge | Unpleasantness of Percept | Loss of Sense of Self | Annoyance |
|---|---|---|---|---|---|---|---|---|---|---|---|---|---|---|---|---|---|---|
| Constant Awareness | 0 | | | | | | | | | | | | | | | | | |
| Emotional Reaction | 11 | 0 | | | | | | | | | | | | | | | | |
| Loss of Peace | 10.099505 | 7.1414284 | 0 | | | | | | | | | | | | | | | |
| Loss of Quiet | 12.369317 | 10.488088 | 9.2195445 | 0 | | | | | | | | | | | | | | |
| Emotional Consequences | 11.61895 | 9.1651514 | 7.8102497 | 10.677078 | 0 | | | | | | | | | | | | | |
| Feeling Imperfect | 12.206556 | 9.8994949 | 8.4261498 | 11.224972 | 10.099505 | 0 | | | | | | | | | | | | |
| Inability to Concentrate | 12.489996 | 10.535654 | 9.486833 | 11.61895 | 11 | 11.445523 | 0 | | | | | | | | | | | |
| Loss of Control | 11.661904 | 9.539392 | 7.8740079 | 10.908712 | 9.7467943 | 9.6436508 | 11.135529 | 0 | | | | | | | | | | |
| Fear | 13.601471 | 11.224972 | 10.630146 | 12.884099 | 12 | 12.083046 | 13.076697 | 11.61895 | 0 | | | | | | | | | |
| Effect on Listening | 11.575837 | 8.8881944 | 7.6157731 | 10.535654 | 9.3273791 | 10.049876 | 10.677078 | 9.591663 | 11.532563 | 0 | | | | | | | | |
| Quality of Life | 14.247807 | 12.328828 | 11.18034 | 13.190906 | 12.165525 | 12.806248 | 13.304135 | 12.4499 | 14.142136 | 12.041595 | 0 | | | | | | | |
| Physical Effects of Tinnitus | 10.440307 | 7.4833148 | 5.9160798 | 9.486833 | 8.1240384 | 8.9442719 | 9.8488578 | 8.5440037 | 10.954451 | 7.9372539 | 11.489125 | 0 | | | | | | |
| Intrusiveness | 10.440307 | 7.6157731 | 5.7445626 | 9.1651514 | 8 | 8.8317609 | 9.6436508 | 7.9372539 | 10.488088 | 7.9372539 | 11.224972 | 6.3245553 | 0 | | | | | |
| Effect on Sleep | 11.575837 | 9 | 7.7459667 | 10.630146 | 9.539392 | 10.246951 | 10.77033 | 9.797959 | 11.532563 | 9.486833 | 12.4499 | 7.9372539 | 8.0622577 | 0 | | | | |
| Need for Knowledge | 10.488088 | 7.2801099 | 5.4772256 | 9.3273791 | 7.9372539 | 8.660254 | 9.591663 | 8.1240384 | 10.440307 | 7.7459667 | 11.357817 | 6.0827625 | 5.9160798 | 7.8740079 | 0 | | | |

| | | | | | | | | | | | | | | | | | | |
|---|---|---|---|---|---|---|---|---|---|---|---|---|---|---|---|---|---|---|
| Unpleasantness of Percept | 10.488088 | 7.8102497 | 6 | 9.4339811 | 8.1853528 | 8.8881944 | 9.6953597 | 8.2462113 | 10.816654 | 8.1240384 | 11.532563 | 6.5574385 | 6.4031242 | 8.1240384 | 6 | 0 | | |
| Loss of Sense of Self | 11.090537 | 8.3666003 | 6.7082039 | 9.8994949 | 8.8317609 | 9.2736185 | 10.049876 | 9.1104336 | 11.313708 | 8.5440037 | 11.916375 | 7.2111026 | 6.9282032 | 8.7749644 | 6.8556546 | 7.2801099 | 0 | |
| Annoyance | 11.789826 | 11.135529 | 9.8488578 | 11.916375 | 11.045361 | 11.916375 | 12.288206 | 11.269428 | 13.56466 | 11.090537 | 13.856406 | 10 | 10.099505 | 11.090537 | 9.8488578 | 10.34408 | 10.583005 | 0 |
